# ATF4 inhibits tumor development and mediates p-GCN2/ASNS upregulation in colon cancer

**DOI:** 10.1038/s41598-024-63895-y

**Published:** 2024-06-06

**Authors:** Jiawei Chen, Xiaopeng Huang, Shuai Zhang, Xiaodong Zhu

**Affiliations:** 1 Department of Radiation Oncology, Guangxi Medical University Cancer Hospital, No. 71 Hedi Road, Qingxiu District, Nanning, 530021 Guangxi China; 2 Department of Radiation Oncology, Hainan General Hospital, Hainan Affiliated Hospital of Hainan Medical University, Haikou, Hainan China; 3 Department of Oncology, Wuming Hospital of Guangxi Medical University, Nanning, Guangxi China; 4 Key Laboratory of Early Prevention and Treatment for Regional High Frequency Tumor, Guangxi Medical University, Ministry of Education, Nanning, Guangxi China; 5 Guangxi Key Laboratory of Early Prevention and Treatment for Regional High Frequency Tumor, Nanning, Guangxi China

**Keywords:** Colon cancer, ATF4, p-GCN2/ASNS, Invasion, Apoptosis, Gastrointestinal cancer, Molecular biology

## Abstract

Colon cancer (CC) is a highly malignant tumor with a high incidence and poor prognosis. This study aimed to explore the function and molecular mechanisms of activating transcription factor 4 (ATF4) in CC. The expression levels of ATF4, GCN2, and ASNS in CC tissues were measured using immunohistochemistry (IHC) and reverse transcription quantitative PCR (RT-qPCR). Cell counting kit-8 (CCK-8), clone formation, transwell, and flow cytometry assays were conducted to assess cell viability, clonogenicity, migration, invasion, cell cycle, and apoptosis, respectively, in the ATF4 knockdown and overexpression SW480 cell lines. The effect of ATF4 on the expression of GCN2 and ASNS was detected using RT-qPCR, Chip-qPCR, and western blotting. ATF4, GCN2, and ASNS were expressed at low levels in CC tissues, and all had a significant negative correlation with tumor diameter. ATF4 knockdown promoted cell proliferation, invasion, and S-phase cell cycle and inhibited apoptosis in SW480 cells. In contrast, ATF4 overexpression had the opposite effect. Furthermore, ATF4 overexpression enhanced ATF4 binding to the ASNS promoter region. ATF4 knockdown significantly inhibited the expression of p-GCN2 and ASNS, whereas ATF4 overexpression significantly upregulated their expression. ATF4 inhibited CC cell viability, clone formation ability, migration, and invasion and promoted apoptosis, possibly by regulating the expression of p-GCN2 and ASNS. Our study provides a novel potential therapeutic target for the treatment of CC.

## Introduction

Colon cancer (CC) is the most prevalent malignant tumor globally, contributing to an estimated 700,000 deaths annually and representing 5.8% of all cancer-related fatalities^1^. To date, several therapies, including surgery, radiotherapy, chemotherapy, and multidisciplinary combination therapy, are the mainstay treatments for CC, which have reduced the morbidity and mortality of patients with CC^2^. However, most diagnosed cases of CC are in the advanced stages, and approximately 60% of patients with CC are prone to distant metastases, resulting in a poor prognosis^3^. Therefore, there is an urgent need to investigate the underlying molecular mechanisms of CC and identify novel therapeutic targets.

The activating transcription factor (ATF)/cAMP response element-binding protein (CREB) family contains a conserved basic leucine-zipper domain^4^. To date, more than 20 ATF/CREB members have been identified in mammals, with many of them playing crucial roles in tumor progression^5^. Activating transcription factor 4 (ATF4), which belongs to the ATF/CREB family, is a mammalian DNA-binding protein that is extensively expressed in various cell lines^6^. It plays a crucial role in cellular stress responses, particularly in the context of endoplasmic reticulum (ER) stress and amino acid deprivation^7,8^. ATF4 dysregulation has been implicated in various diseases, such as osteoporosis, neurodegeneration, skeletal muscle atrophy, Alzheimer’s disease, and tumors^9–12^. Disruption of ATF4-dependent fructolysis, either through genetic or pharmacological intervention, inhibits growth and colony formation of glioblastoma multiforme cells^13^. Under ER stress induced by thapsigargin and brefeldin A, activation of PERK/eIF2α/ATF4 signaling reduced androgen receptor expression and ATF4 overexpression inhibited triple-negative breast tumor proliferation^14^. Moreover, recent studies have demonstrated that ATF4 is involved in CC development. PERK-ATF4 pathway activation contributes to enhanced chemoresistance of CC cells^15^. ATF2/ATF4 regulates Wnt target genes in CC cells in a β-catenin-independent manner, thereby influencing CC progression^16^. STIM2 deficiency stimulates cMyc and the PERK/ATF4 branch of the ER stress pathway in an Orai-independent manner, thereby promoting CC cell growth and metastasis^17^. However, the function of ATF4 and its underlying molecular mechanisms in CC remain unclear and warrant further investigation.

Therefore, in the present study, we focused on the effects of ATF4 on CC cell functions, including cell viability, clone formation, cell cycle, migration, invasion, and apoptosis. This study also aimed to explore the correlation among the expression of ATF4, GCN2, and ASNS in CC. This study enhances our understanding of CC pathogenesis and offers potential approaches for clinical treatment.

## Materials and methods

### Human participants and tissue specimens

Five patients diagnosed with CC were recruited from Guangxi Medical University Cancer Hospital between May 2022 and January 2023. After obtaining informed consent from each patient, five whole-section samples of CC tissues and five adjacent non-cancerous tissues (ANCT) were harvested. All patients were free of infectious diseases and had not received any treatment before surgery. This study was conducted in accordance with the Second Helsinki Declaration and approved by the Ethics Committee of Guangxi Medical University Cancer Hospital (Ethics number: 2022J181). The clinicopathological characteristics of the patients are shown in Supplementary Table 1.

### Immunohistochemistry (IHC) analysis

Clinical CC tumors and paracancerous tissues were collected and fixed in 4% paraformaldehyde solution. After dehydration with a gradient of 50%, 70%, 80%, 90%, and 95% anhydrous ethanol, the tissues were immersed in a 1:1 mixture of anhydrous ethanol and xylene for half an hour and then made transparent in a pure xylene solution. Following paraffin embedding, CC tumor and adjacent non-cancerous tissues were subsequently sectioned into 4-μm slices. Following rehydration, the sections were subjected to microwave treatment in a citrate buffer to facilitate antigen recovery. Endogenous peroxidase activity was inhibited by 3% H_2_O_2_. Subsequently, the sections were incubated with a 5% BSA solution for 30 min, followed by incubation with the primary anti-ATF4 (dilution 1:800; Affinity Biosciences, Jiangsu, China; DF6008), anti-p-GCN (dilution 1:800; Affinity Biosciences; AF8154), AND anti-ASNS (dilution 1:800; Affinity Biosciences; DF7398) at 4 °C overnight and then with the corresponding secondary antibody. Treatment with DAB (3,3′-diaminobenzidine) was applied to the sections, and hematoxylin served as the counterstain. Finally, the staining was observed using an Olympus BX50F microscope (Olympus, Tokyo, Japan).

### Cell culture

The human CC line SW480 was procured from Icell Bioscience Biotechnology Co., Ltd. (Shanghai, China). All cell lines were maintained in DMEM supplemented with 10% FBS, 100 U/mL penicillin, and 100 mg/mL streptomycin at 37 °C with 5% CO_2_.

### Cell transfection

Small-interfering RNAs (siRNAs) were used to downregulate ATF4 in SW480 cells, and the pcDNA3.1-ATF4 overexpression plasmid was used to upregulate ATF4 expression. The si-control (si-NC), si-ATF4, pcDNA3.1-ATF4plasmid, and control plasmid (oe-NC) used for transfection were obtained from Suzhou GenePharma Co., Ltd. (Suzhou, China). SW480 cells were seeded at a density of 1 × 10^5^ cells/well in 6-well plates. When the cells reached 70–80% confluence, cell transfection was performed using Lipofectamine 3000 (Thermo Fisher Scientific, Massachusetts, USA) according to the manufacturer's instructions. Briefly, the first tube of transfection reagent was prepared by taking 5 μL of Opti-MEM and adding 0.25 μL of Lipofectamine 3000 solution. For the second tube of transfection reagent, 5 μL of Opti-MEM, 5 pM of the corresponding plasmids, and 0.25 μL of Lipofectamine 3000 solution were combined. The two solutions were mixed at a ratio of 1:1 and allowed to stand for 15 min at room temperature. The mixture was added to the corresponding culture dishes at 10 μL/well and then placed in a 37 °C and 5% CO_2_ incubator. Cells were collected 48 h post-transfection for further experiments, and transfection efficiency was determined via reverse transcription quantitative polymerase chain reaction (RT-qPCR). Untreated SW480 cells were used as controls.

### RT-qPCR

Total RNA was isolated from SW480 cells using TRIZOL reagent (Solarbio, Beijing, China; R1200). The Hifair^®^ III 1st Strand complementary DNA (cDNA)Synthesis SuperMix kit (Yeasen, Shanghai, China) was employed to synthesize complementary DNA (cDNA) using 2 μg of isolated RNA. RT-qPCR analysis was conducted on cDNA using an ABI 7500 RT-PCR system (Thermo Fisher Scientific) with SYBR Premix Ex Taq (Takara, Dalian, China), in accordance with the manufacturer's guidelines. The mRNA expression levels were normalized to GAPDH and evaluated using the comparative 2^−ΔΔCT^ method. The primers used for qRT-PCR are listed in Supplementary Table 2.

### Cell counting kit-8 (CCK-8) assay

Cell viability was assessed using the CCK-8 assay. Transfected SW480 cells were seeded into 96-well plates at a density of 1 × 10^5^ cells/well^18^. Following incubation for 24, 48, and 72 h in the incubator at 37 °C containing 5% CO_2_, the initial medium was removed and replaced with new DMEM. To each well, 10 μL of CCK-8 assay solution was added. After 2 h of incubation at 37 °C, the optical density (OD) value of the cells at 450 nm was evaluated using a microplate reader (DALB, Shanghai, China).

### Clone formation assay

For the clone formation assay, 4 × 10^2^ SW480 cells were cultivated in 2 mL of complete medium in 6-well plates. The cells were cultured for 7 days and observed regularly. The culture was terminated based on the appearance of visible clones in the culture dish. Subsequently, 2 mL 4% paraformaldehyde was added to fix the cells for 10 min, followed by staining with 2 mL 5% crystal violet for 8 min. The Petri dishes were inverted, and a transparent film with a grid was superimposed to count the number of clones with the naked eye, which was then recorded. The rate of clone formation was calculated as follows: number of clones/number of inoculated cells × 100.

### Cell cycle assay

Cell cycle assays were conducted using a Cell Cycle Assay kit (7sea biotech, Shanghai, China; C005-200), according to the manufacturer's instructions. Briefly, after 24 h of cultivation, the transfected SW480 cells were centrifuged at 1000 rpm for 5 min. Subsequently, 1 mL of pre-cooled 70% alcohol was added to gently resuspend the cells, and the cells were fixed at 4 °C overnight. After 30 min of incubation in the dark with a solution containing 100 μg/mL of propidium iodide (PI), the cells were quantified utilizing a FACScan flow cytometer (Becton, Dickinson and Company, New Jersey, USA). CytExpert V2.3.0.84 software (Beckman Coulter, USA) was used to assess cell cycle distribution.

### Transwell migration and invasion assay

The density of SW480 cells was adjusted to 2.5 × 10^5^ cells/mL using serum-free medium. In the lower chamber (bottom of the 24-well plate), 700 μL of medium containing 10% serum was added, and 200 μL of cell suspension was added to the upper chamber. The cells were cultured in an incubator for 24 h. Then, 700 μL 4% paraformaldehyde was added to the upper chamber, and the cells were fixed at room temperature for 10 min. Next, the cells were fixed with 100% methanol for 20 min and stained with 700 μL of 2.5% crystal violet solution for 15 min. The cells located on the surface of the membrane in the lower part of the upper chamber were carefully wiped with a wet cotton swab, and the chambers were thoroughly dried and photographed using an inverted microscope (Olympus) at 100 × magnification. The invasion investigations differed from the migration studies in that the membranes were covered with Matrigel^®^ (Corning, New York, USA) for 24 h prior to the examinations.

### Apoptosis assay

Apoptosis assays were performed using an Annexin V-FITC/PI double-stained cell apoptosis detection kit (7sea biotech; A005-3). After washing the cells with cold PBS, 400 μL of 1 × binding buffer was added to gently resuspend the cells. Next, 5 μL of Annexin V-FITC was added, and the mixture was incubated for 15 min at room temperature in the dark. Subsequently, 10 μL of PI staining solution was added, and the samples were incubated on an ice bath for 5 min in the dark. A FACScan flow cytometer (Becton, Dickinson and Company) was used to stain cells for apoptosis.

### Western blotting

Complete proteins were harvested using radioimmunoprecipitation assay buffer containing protease inhibitors (Solarbio). After collecting the whole-cell extracts, the protein content was quantified using a bicinchoninic acid kit (Solarbio). The proteins were separated on a sodium dodecyl sulfate-sulfate–polyacrylamide gel and transferred onto polyvinylidene difluoride membranes (Roche, Basel, Switzerland) at 200 mA for 90 min. The membranes were blocked at room temperature using TBST containing 5% milk and then incubated with primary antibodies for 2 h at room temperature, including anti-p-GCN (dilution 1:1000; Affinity Biosciences; AF8154), anti-GCN (dilution 1:1000; Affinity Biosciences; DF7801), anti-ASNS (dilution 1:1000; Affinity Biosciences; DF7398), and anti-GAPDH (dilution 1:1000; Affinity Biosciences; AF7021). Subsequently, the membranes were incubated with a HRP-labeled secondary antibody (dilution 1:5000, Bioss, Beijing, China; bs-0295G-HRP) at room temperature for 2 h. Protein bands were visualized using ECL reagent (Amersham, Little Chalfont, UK).

### Chip-qPCR

Approximately 5 × 10^6^ cells were fixed in 1% formaldehyde for 10 min at room temperature. The fixed cells were lysed using a sonicator (Diagenode, Liège, Belgium; UCD-300) for (30 s + 30 s) sonication for 28 cycles. Immunoprecipitation (IP) was performed using ATF4 antibody and IgG antibodies. PCR amplification of the precipitated DNA was performed. Supplementary Table 3 shows the primer sequences used for the chip-qPCR assays.

### Statistical analyses

Statistical analysis were carried out using GraphPad Prism 7.0 (GraphPad Software, USA), and the results are depicted as means ± standard deviation (SD). The Pearson correlation coefficient was used to evaluate the correlation among ATF4, GCN2, and ASNS expression and tumor diameter. Student’s t-test was used to compare two groups, whereas Tukey's test in conjunction with one-way analysis of variance was employed to assess differences among multiple groups. Statistical significance was set at a threshold of p < 0.05.

## Results

### ATF4, p-GCN2, and ASNS were lowly expressed in CC

**Figure 1:**
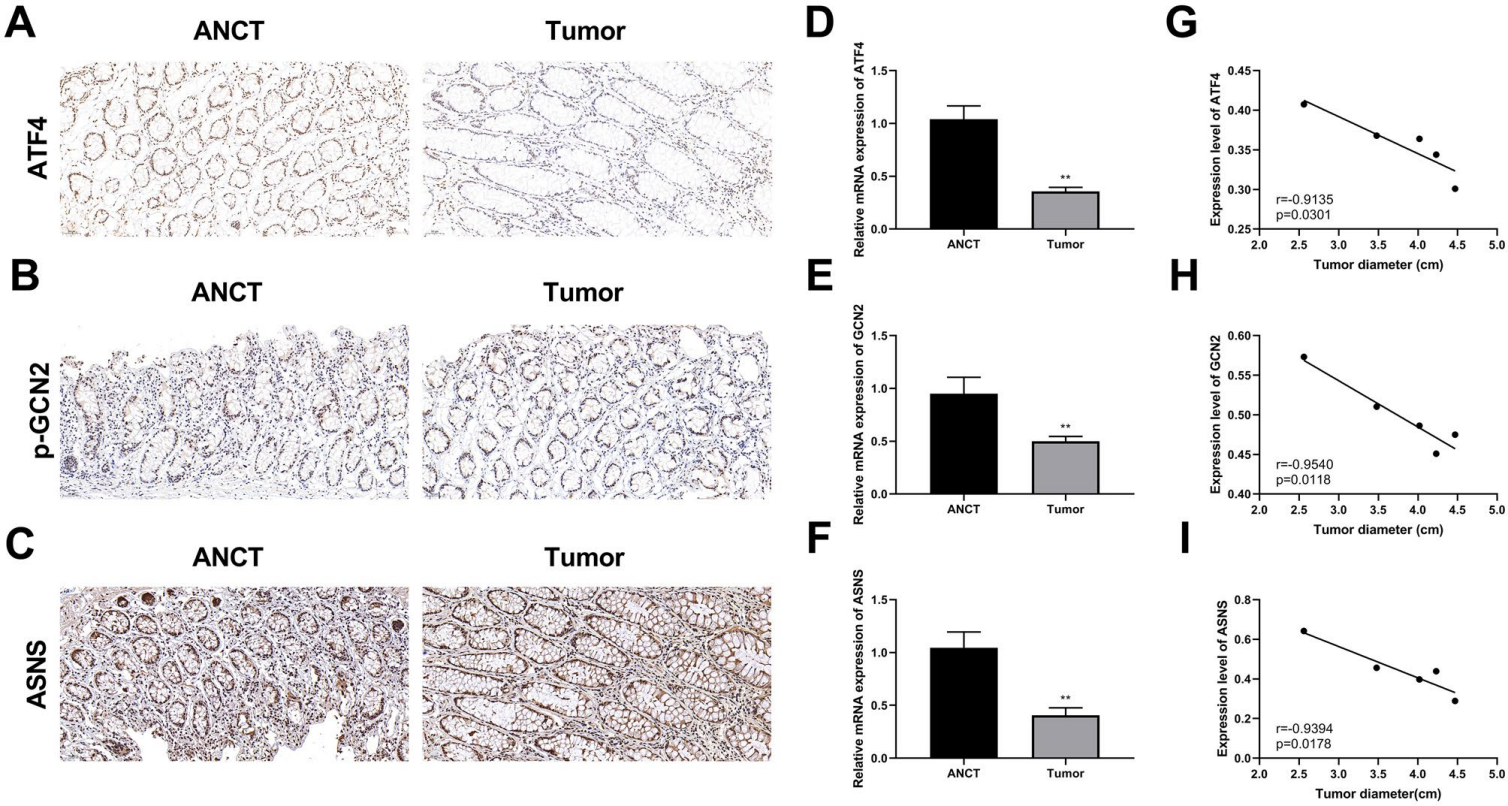
ATF4, p-GCN2, and ASNS are lowly expressed in CC. (**A**) IHC staining was employed to measure ATF4 expression. (**B**) IHC results of p-GCN2. (**C**) IHC results of ASNS. Magnification: 200×; scale bar: 50 μm. (**D**–**F**) The mRNA levels of ATF4 (**D**), GCN2 (**E**), and ASNS (**F**) in CC tumor tissues and adjacent non-cancerous tissues (ANCT) were measured by RT-qPCR. (**G**–**I**) The relationship between the expressions of ATF4 (**G**), GCN2 (**H**), and ASNS (**I**) and the tumor diameter. **p < 0.01 vs. ANCT group.

A previous study has revealed that GCN2 activation induces the translation and transcription of ATF4-related regulatory genes. ATF4 is a key transcription factor that regulates ASNS^19^. Consequently, we explored the expression of ATF4, p-GCN2, and ASNS in the CC tissues. IHC results showed that the proportion of positive cells for ATF4 (Fig. 1A), p-GCN2 (Fig. 1B), and ASNS (Fig. 1C) were significantly lower in tumor tissues than in paracancerous tissues. Similarly, RT-qPCR showed that the mRNA levels of ATF4, GCN2, and ASNS were notably reduced in CC tissues compared to those in ANCT tissues (Fig. 1D–F). Moreover, there was a significant negative correlation between the expression levels of ATF4, GCN2, and ASNS and tumor diameter in patients with CC (Fig. 1G–I). These results suggest that ATF4 GCN2, and ASNS are involved in CC progression.

### ATF4 inhibited CC cell viability, S-phase cell cycle, and clonogenicity

**Figure 2:**
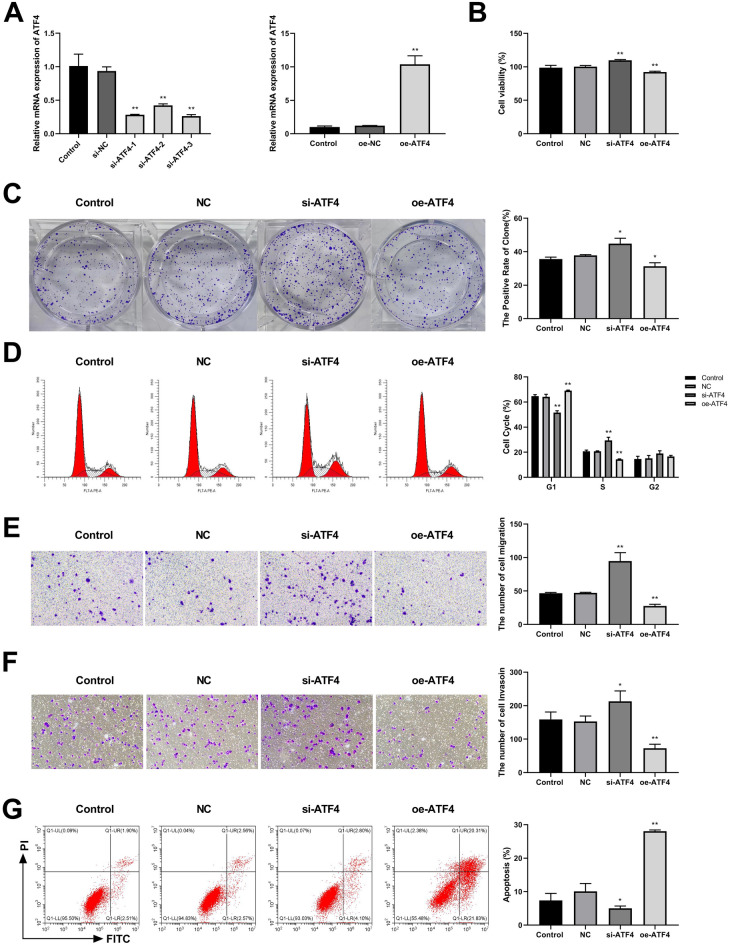
ATF4 impedes the progression of CC. (**A**) Transfection efficiency in SW480 cell line, both silenced and overexpressed for ATF4, was assessed using RT-qPCR. **p < 0.01 vs. si-NC or oe-NC group. (**B**) CCK8 was used to assess the effect of ATF4 silencing and overexpression on SW480 cell proliferation. (**C**) The influence of ATF4 silencing and overexpression on CC cell clone-forming ability was examined using clone-forming assay. (**D**) After treated with si-ATF4 and oe-ATF4, cell cycle of SW480 cells was detected by flow cytometry. (**E**,**F**) The migration (**E**) and invasion (**F**) of SW480 cells were assessed by Transwell assays in control, NC, si-ATF4, and oe-ATF4 groups. (**G**) Flow cytometry was employed to measure the SW480 cell apoptosis rate after si-ATF4 or oe-ATF4 transfection. *p < 0.05; **p < 0.01 vs. NC group.

To explore the role of ATF4 in CC, we constructed the ATF4 silencing and overexpression SW480 cell line. RT-qPCR results showed that there was no significant difference in the expression of ATF4 between the control and ATF4 silencing or overexpression negative controls. There was a significant reduction in ATF4 expression in the si-ATF4-1, si-ATF4-2, and si-ATF4-3 groups compared with that in the si-NC group. As si-ATF4-3 showed higher and more stable knockdown efficiency comparison to si-ATF4-1 and si-ATF4-2, it was chosen for subsequent experiments. Additionally, ATF4 expression was notably elevated in the oe-ATF4 group compared to that in the oe-NC group (Fig. 2A). Subsequently, cell viability was evaluated using a CCK8 assay. These findings indicate that ATF4 silencing enhanced cell viability, whereas ATF4 overexpression inhibited cell viability (Fig. 2B). The colony formation assay revealed that SW480 cells produced larger and more colonies in the si-ATF4 group than in the NC group. Conversely, SW480 cells formed smaller and fewer colonies in the oe-ATF4 group comparison to NC group (Fig. 2C). Furthermore, ATF4 knockdown caused G1-phase arrest and S-phase accumulation in SW480 cells. ATF4 overexpression, in contrast, promoted the G1 phase and inhibited the S phase cell cycle (Fig. 2D). Taken together, our data demonstrate that ATF4 suppresses the proliferation, S-phase cell cycle, and colony formation in CC cells.

### ATF4 suppressed CC cell migration and invasion and promoted apoptosis

Subsequently, we evaluated the effect of ATF4 on CC cell migration and invasion using Transwell assays. No significant differences were observed in the number of migrating and invading cells between the control and NC groups. Relative to the NC group, the migration and invasion of SW480 cells significantly increased in the si-ATF4 group, whereas they notably decreased in the oe-ATF4 group (Fig. 2E,F). Apoptosis was evaluated using flow cytometry. The results showed that there was no significant difference in the rate of apoptosis between the NC and control groups. After transfection with si-ATF4, the apoptotic rate of SW480 cells was markedly reduced. Conversely, treatment with oe-ATF4 significantly promoted the apoptosis of SW480 cells (Fig. 2G). Collectively, ATF4 may inhibit CC cell migration and invasion, while promoting apoptosis.

### ATF4 promoted p-GCN and ASNS expression

**Figure 3:**
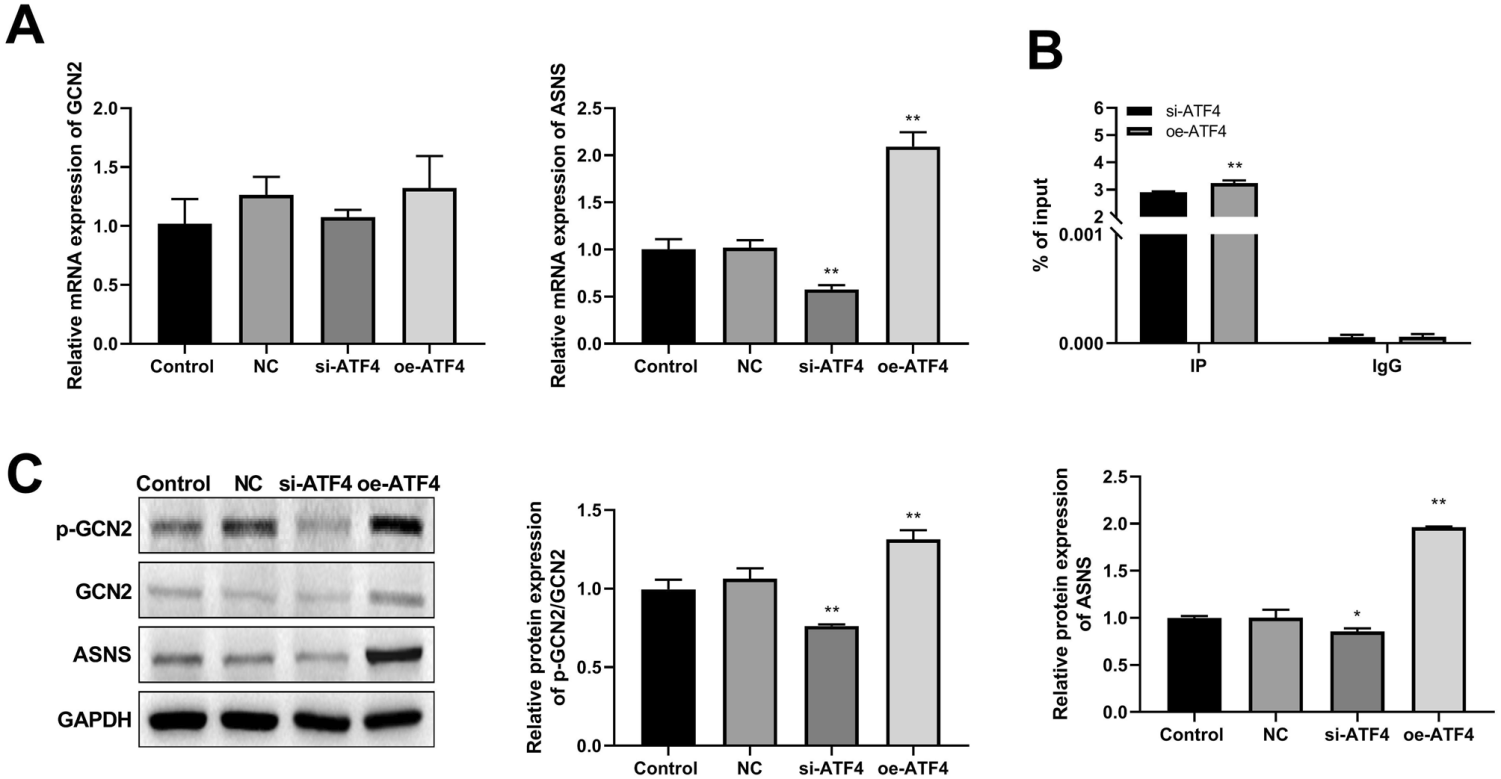
ATF4 promotes p-GCN and ASNS expression. (**A**) The effect of ATF4 knockdown and overexpression on GCN and ASNS mRNA expressions was evaluated by RT-qPCR. (**B**) ATF4 binding to the ASNS promoter was examined by ChIP-qPCR. (**C**) Western blot was performed to evaluate the impact of ATF4 silencing and overexpression on the p-GCN and ASNS protein expressions. *p < 0.05; **p < 0.01 vs. NC group.

We investigated the effects of ATF4 silencing and overexpression on the expression of GCN and ASNS using RT-qPCR. These results demonstrated that ATF4 silencing and overexpression had no significant effect on GCN2 expression. ATF4 silencing markedly inhibited ASNS expression, whereas ATF4 overexpression significantly promoted ASNS expression (Fig. 3A). Furthermore, the ChIP-qPCR results demonstrated that ATF4 bound to the ASNS promoter upon ATF4 overexpression, enhancing its binding activity (Fig. 3B). Western blotting revealed that ATF4 silencing notably suppressed GCN protein phosphorylation and ASNS protein expression. Conversely, ATF4 overexpression significantly increased the protein levels of p-GCN and ASNS (Fig. 3C). Thus, ATF4 may affect CC development by regulating the protein expression of p-GCN and ASNS.

## Discussion

CC is a malignancy with a high incidence rate and poses a substantial threat to human health^3^. The development and progression of CC are regulated by various oncogenes and tumor suppressor genes^20^. Advancements in modern biological techniques and molecular investigations have revealed numerous genes associated with CC, including KRAS, BRAF, PI3K, and p53, which play crucial roles in different stages of CC^20^. However, CC pathogenesis of CC is highly complex. Further investigations are essential to develop more effective and targeted therapeutic strategies for treating CC. In this study, we explored the role and potential molecular mechanisms of action of ATF4 in CC. IHC staining showed a significant decrease in the expression of ATF4 in CC tissues. ATF4 silencing significantly promoted cell viability, clone formation, S-phase cell cycle, migration, and invasion, while inhibiting apoptosis. The overexpression of ATF4 had the opposite effect. Mechanistically, ATF4 markedly promoted the expression of p-GCN and ASNS.

ATF4 is a stress-induced gene activated by hypoxia, endoplasmic reticulum stress, and amino acid deprivation^21–23^. ATF4 is involved in amino acid transport, metabolism, protein homeostasis, and protection against oxidative stress. It also regulates proliferation, apoptosis, cell cycle, and senescence in tumors^7^. Previous studies have revealed that ATF4 overexpression reduces migration, invasiveness, proliferation, and epithelial-mesenchymal transition in triple-negative breast cancer cells by regulating the SMAD2/3/4 and mTORC2 signaling pathways^24^. By upregulating the expression of ATF4, LXRβ activation reduces proliferation and increases chemosensitivity of gastric cancer cells^25^. Furthermore, Withaferin A induces G2/M arrest in glioblastoma cells through the ATF4-ATF3-CHOP axis^26^. In this study, we found that ATF4 overexpression significantly inhibited proliferation, S-phase cell cycle, migration, and invasion, while promoting apoptosis in SW480 cells. In contrast, ATF4 knockdown had the opposite effect. Several studies have indicated that ATF4 may play an antitumor role in CC. The increased expression of ERS-related proteins (including GRP78/BIP, BAX, and PERK/elF2α/ATF4/CHOP) was induced by overexpression of miR-451a or silencing of BAP31, which inhibits the proliferation of colorectal cancer cells^27^. TSPYL5 overexpression promotes the expression of caspase-1, caspase-3, Bax, ATF4, and CHOP proteins, thereby suppressing the proliferation, migration, and invasion of CC tumor cells^28^. These findings suggest that ATF4 may play a role in inhibiting tumor proliferation and growth in CC. However, another study showed that activation of the PERK-ATF4 pathway enhances chemoresistance in CC cells^15^. Thus, the role of ATF4 in CC is complex and it may play a dual role. ATF4 promotes apoptosis under persistent stress^7^. Activated ATF4 drives CHOP during long-term ER stress, causing an unfolded protein response in the terminal apoptotic pathway in plasma cells^29^. In esophageal squamous cell carcinoma, FAM175B promotes apoptosis by inhibiting ATF4 ubiquitination^30^. Through the PERK/ATF4/CHOP pathway activation, VPS34-IN1 induces ER + breast cancer cell apoptosis^31^. In CC, the activation of TAp73α by ER stress facilitates the apoptosis of tumor cells via the PERK-ATF4 signaling pathway^32^. In addition, through oxidative stress and the PERK/eIF2α/ATF4/CHOP axis, naringin, NF-κB inhibition, and endoplasmic reticulum stress together cause apoptotic cell death in HT29 CC cells^33^. The results of this study illustrate the ability of ATF4 to promote apoptosis in SW480 CC cells, which is consistent with previous investigations.

Amino acids play a crucial role in cancer metabolism, serving as both components and sensors in signaling pathways that regulate major biological processes^34^. Dysregulated sensing of amino acids promotes the growth of colorectal cancer and induces metabolic reprogramming, ultimately leading to chemoresistance^35^. Many amino acid-metabolizing enzymes play important roles in CC development. JPH203 can inhibit the function of LAT1 through a preincubation effect, providing a novel approach for the treatment of CC^9^. The cystine transporter SLC7A11 is involved in CC progression by regulating ferroptosis^36,37^. In our investigation, we found that ATF4 overexpression induced enhanced binding activity of ATF4 to the ASNS promoter region. Moreover, ATF4 promoted p-GCN and ASNS protein expression. ASNS is a single gene encoded in the mammalian genome that facilitates the transformation of aspartate into asparagine^38^. GCN2 regulates transcription and translation in response to availability of nutrition^39^. ASNS activity is tightly regulated in response to cellular stress. Activation of the GCN2-eIF2-ATF4 signaling pathway, which results in increased ASNS expression, seems to be part of the solid tumor adaptation to conditions, such as nutrient deprivation and/or hypoxia^40^. In gastric and esophageal cancers, ATF4 interacts with ASNS to influence tumor progression^41,42^. AMPK and GCN2-ATF4 are involved in CC HCT116 cells cell mitochondrial repression^43^. Moreover, ATF4 knockdown drastically decrease the levels of ASNS, whereas ASNS overexpression inhibits the growth and enhances the survival of ATF4 knockdown tumor cells^44^. Therefore, we hypothesized that the ATF4/p-GCN2/ASNS axis plays a crucial role in maintaining metabolic homeostasis in tumor cells, making it a novel and attractive target for antitumor approaches.

## Conclusion

In this study, we observed a reduction in the expression of ATF4, GCN2, and ASNS in CC tissues. The expression levels of ATF4, GCN2, and ASNS were significantly and negatively correlated with CC tumor diameter. ATF4 silencing markedly enhanced cell viability, clone formation, S-phase cell cycle progression, migration, and invasion while suppressing apoptosis. Conversely, ATF4 overexpression had the opposite effect. Moreover, ATF4 upregulated the protein expression of p-GCN and ASNS. This study provides novel perspectives on CC management.

### Supplementary Information

Supplementary Tables.

Supplementary Information.

## Data Availability

The data and materials supporting the findings of this study are available from the corresponding authors upon request.
