# Supplementary material for: ATF4 inhibits tumor development and mediates p-GCN2/ASNS upregulation in colon cancer
Source: Sci Rep. 2024 Jun 6;14:13042. doi: 10.1038/s41598-024-63895-y (PMC11156644; doi:10.1038/s41598-024-63895-y)
Supplement: Supplementary file 1 — Supplementary Tables. [file 41598_2024_63895_MOESM1_ESM.docx]

Supplementary table 1 Clinicopathological characteristics of the patients

| Patient number | ID | Age (years) | Gender | TNM stage | Clinical stage | Tumor type | Tumor diameter (cm) |
| --- | --- | --- | --- | --- | --- | --- | --- |
| 1 | PA2127821 | 45 | male | T2N0M0 | I | Adenocarcinoma | 2.56 |
| 2 | PA2127807 | 56 | female | T3N0M0 | II | Adenocarcinoma | 3.48 |
| 3 | PA2127371 | 65 | male | T3N0M0 | II | Adenocarcinoma | 4.02 |
| 4 | PA2127373 | 72 | female | T3N1M0 | III | Adenocarcinoma | 4.23 |
| 5 | PA2127452 | 78 | male | T4N1M0 | III | Adenocarcinoma | 4.47 |

**Supplementary table** **2** Primer sequences in RT-qPCR

| Genes | Primer sequences | | Primer fragment size (bp) |
| --- | --- | --- | --- |
| h-ATF4 | F | ACGTTGGATGACACTTGTGATCTCTT | 26 |
|  | R | TGTAGTCTGGCTTCCTATCTCCTTCA | 26 |
| h-GCN2 | F | GTCCTTCCTGCCTGCTTCAGATTC | 24 |
|  | R | CCCTGGTCAATGGTGTCTCGTAAAG | 25 |
| h-ASNS | F | CTTGGTTGCTGCCACTCTGTTGA | 23 |
|  | R | CCACGCTATCTGTGTTCTTCCGAAT | 25 |
| GAPDH(h) | F | TCAAGAAGGTGGTGAAGCAGG | 21 |
|  | R | GCGTCAAAGGTGGAGGAGTG | 20 |

**Supplementary table 3** Primer sequences in Chip-qPCR

| Genes | | Primer sequences | | Primer fragment size (bp) | |
| --- | --- | --- | --- | --- | --- |
| ASNS-F | | TCTGCCTGAACACCCTCCACTG | | 124bp | |
| ASNS-R | | ACAGGAGGCTCAGCAGGTCTTG | |  | |
| GAPDH | | AAAAGCGGGGAGAAAGTAGG | | 212bp | |
| GAPDH | | AAGAAGATGCGGCTGACTGT | |  | |
