# Supplementary figures and images for: ATF4 inhibits tumor development and mediates p-GCN2/ASNS upregulation in colon cancer
Source: Sci Rep. 2024 Jun 6;14:13042. doi: 10.1038/s41598-024-63895-y (PMC11156644; doi:10.1038/s41598-024-63895-y)

Fig.3C-p-GCN2

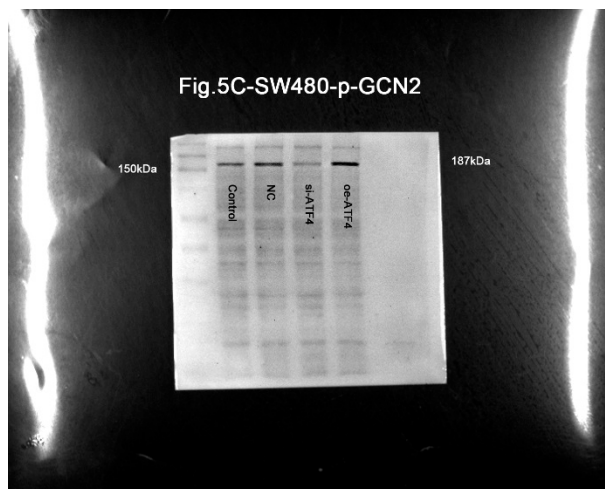

Fig.3C-GCN2

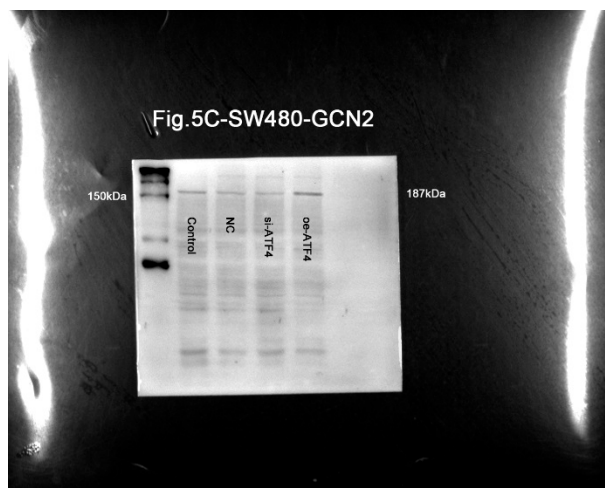

Fig.3C-ASNS

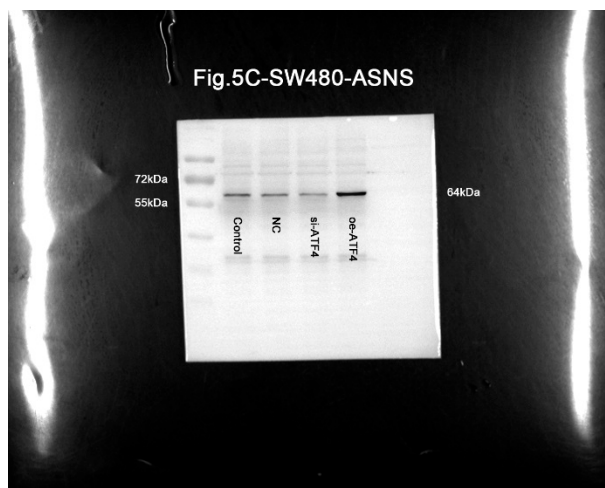

Fig.3C-GAPDH

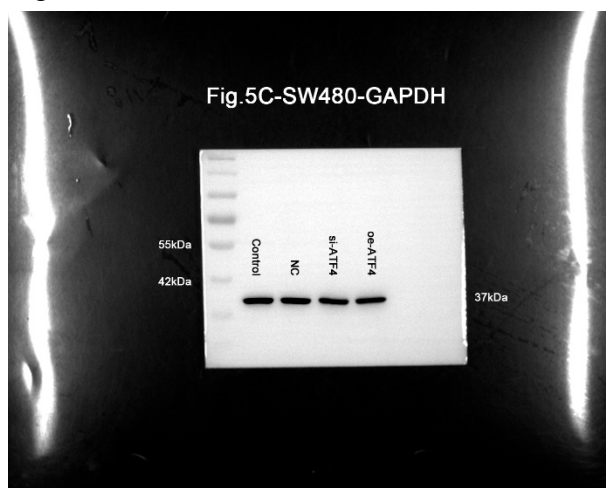

Supplement: Supplementary file 2 — Supplementary Information. [file 41598_2024_63895_MOESM2_ESM.pdf]
